# Laser Spot Detection Based on Reaction Diffusion

**DOI:** 10.3390/s16030315

**Published:** 2016-03-01

**Authors:** Alejandro Vázquez-Otero, Danila Khikhlukha, J. M. Solano-Altamirano, Raquel Dormido, Natividad Duro

**Affiliations:** 1 Department of Computer Sciences and Automatic Control, UNED, C/ Juan del Rosal, 16, 28040 Madrid, Spain; raquel@dia.uned.es (R.D.); nduro@dia.uned.es (N.D.); 2 ELI Beamlines, Institute of Physics ASCR, Na Slovance 2, Prague 8, 18221, Czech Republic; Danila.Khikhlukha@eli-beams.eu (D.K.); jmanuel.solano@correo.buap.mx (J.M.S.-A.); 3 Facultad de Ciencias Químicas, Benemérita Universidad Autónoma de Puebla, 14 Sur y Av. San Claudio, Col. San Manuel, 72530 Puebla, Mexico

**Keywords:** reaction diffusion models, Turing patterns, reaction diffusion computers, reaction diffusion computation, Fitzhugh-Nagumo model, laser spot position, laser spot detection, laser beam detection

## Abstract

Center-location of a laser spot is a problem of interest when the laser is used for processing and performing measurements. Measurement quality depends on correctly determining the location of the laser spot. Hence, improving and proposing algorithms for the correct location of the spots are fundamental issues in laser-based measurements. In this paper we introduce a Reaction Diffusion (RD) system as the main computational framework for robustly finding laser spot centers. The method presented is compared with a conventional approach for locating laser spots, and the experimental results indicate that RD-based computation generates reliable and precise solutions. These results confirm the flexibility of the new computational paradigm based on RD systems for addressing problems that can be reduced to a set of geometric operations.

## 1. Introduction

Nowadays, self-organization is a prolific area of research that throughout the last century has struggled with developing models to reproduce the complexity found in nature. Reaction Diffusion (RD) system models belong to this class, and they aim to describe chemical reactions, that under some circumstances [1], may exhibit stable pattern formation, or even more rich dynamics [2,3]. All of these interesting properties have already been explored in the context of problem-solving by means of diverse experimental setups [4,5,6,7,8,9,10]. Unlike its experimental counterparts, computational implementations do not rely on energy dissipation, for there is no consumption of chemical species as is required in the *thermodynamics of out-of-equilibrium*. Therefore, it results in the appealing possibility of taking advantage of all the properties of RD models’ dynamics, such as their inherent robustness against noisy data and automatic dismissal of the typical drawbacks of experimental setups. Some of these properties include natural parallelism of the model propagation; for instance, the frontwaves that separate stable states into a bistable configuration propagate with constant velocity, and all possible solutions may be explored in parallel [11,12]. Also, due to its strong nonlinear character, the interactions between these wavefronts produce counterintuitive results. It has been shown that such interactions can be used to encapsulate the underlying logic of different types of geometric operations, and indeed, this has led to the development of a novel computational framework capable of addressing robotics-related problems [13,14,15].

In this paper, the problem of finding the center of a laser spot with high accuracy by means of the aforementioned RD-based computational framework is addressed, extending its range of applicability beyond robotic approaches. Traditionally, this problem is carried out in several steps. The algorithms are diverse, depending on the specific applications (e.g., gaming, laser measurement, laser triangulation method, cutting edge laser physics experiments such as laser plasma, X-ray production, sub-atomic particle creation, and even military applications such as target designation or ammunition guidance—See [16] and references therein). However, two main steps can be clearly stated: first, a spot detection, commonly based on recognizing circular- or ellipsoidal-like shapes combined with color selectivity, followed by the actual computation of the spot center. Here, only the computation of the center is considered, since we are interested in applications where high accuracy is required, such as high-tech laser experiments, wherein the laser spot is known to occupy the majority of the detection system’s output (such as an image provided by a CCD sensor). Contrastingly, in the latter we expect notable aberrations caused by complex production, alignment, and focusing systems. In a research facility such as ELI Beamlines [17], a laser beam is expected to encounter tens of optical devices, such as mirrors (static and deformable), telescopes, lenses, *etc.*, from its production and during its transport towards experimental targets. Therefore, a highly accurate method for performing calibration of the beams is needed. In addition, this method may also be used for post processing of experimental data, wherein accuracy, above speediness, is required for fine-tuning and high-quality experimental characterization of the produced beams.

This paper is organized as follows. The next section provides a brief introduction to the RD systems and a short explanation about how the external (*i.e.*, sensory) information can be introduced into the model dynamics. Section 3 introduces a standard algorithm for the location of a laser spot center, intended to provide a suitable comparison method for the RD-based approach, along with the description of the RD algorithm. The experimental results, as well as a brief discussion of our findings, are presented in Section 4. This paper concludes in Section 5 with a summary of contributions and conclusions, as well as our future directions.

## 2. The Reaction Diffusion System

A detailed description of the relevant information about RD systems and their dynamics, required for the comprehension of this text, can be found in our previous work [15]; nevertheless, for the sake of clarity and self-containment, some fundamental concepts will be reproduced in this and the following sections. A RD model can be written in the simplest way as a system of two equations:

(1) $$\begin{array}{ccc} \dot{u} & = & f\left(u,v\right)+D_{u}▵u\\ \dot{v} & = & g\left(u,v\right)+D_{v}▵v \end{array}$$

Computationally, this system describes the spatio-temporal evolution of both state variables $u=u\left(\vec{x},t\right)$ and $v=v\left(\vec{x},t\right)$ over an integration grid. Originally, the functions $f\left(u,v\right)$ and $g\left(u,v\right)$ represent biochemical aspects of the system, describing how the substances $\left(u,v\right)$ are converted into each other over time. Whilst the so-called diffusive coupling between cells (denoted by the laplacian terms ▵u and ▵v) describes how the substances are spread out in space. The two-variable FitzHugh–Nagumo (FHN) model [18,19] has remained as one of the simplest available RD cell models. Its basic formulation is shaped by the following set of coupled equations, where the particular parameters of the model are denoted as α,β,ϵ, and ϕ:

(2) $$\begin{array}{ccc} \dot{u} & = & \varepsilon \left(u-u^{3}-v+\phi \right)+D_{u}▵u\\ \dot{v} & = & \left(u-\alpha v+\beta \right)+D_{v}▵v \end{array}$$

The first step to analyze its dynamics involves plotting the so-called nullclines, defined as the geometric shape for which $\dot{u}=0$ and $\dot{v}=0$ in the absence of diffusion (*i.e.*, $D_{u}=D_{v}=0$). The nullclines provide information about the asymptotic behaviour of each isolated cell, generating a phase space with information about trajectories and fixed points that may be stable or unstable, depending on the model configuration. Upon that, the dynamics of each cell are governed by a combination of the asymptotic behaviour, defined by its nullcline configuration plus the spatial interactions with other cells due to the diffusive coupling. In addition, an interesting feature of these systems is the possibility of codifying external information in the model dynamics. On the one hand, this can be done through the so-called external forcing, which allows the introduction of binary or even gradient-like information in the computational grid permanently. For example, in [14] this method was used for introducing the environmental information coming from sensors in a RD-based robotics exploration algorithm. On the other hand, the external information can be introduced simply as the initial values for the state variables u,v. In such a case the information is volatile, as it will be overwritten by the spatio-temporal evolution of the model. This last option is the one used throughout this work.

## 3. Laser Spot Centering

### 3.1. Conventional Algorithm for Laser Spot Centroid Calculations

Conventional spot center-finding algorithms are based on matching basic geometrical shapes such as circles or ellipses. The spot is expected to be quite similar to one of these patterns (only small deviations are considered) and the spot shape is fitted as best as possible to the reference shape. For the fitting part of the method, Least-squares, Hough transforms [20], Fourier transforms, Zernike moments [21], Gaussian functions [16], *etc.*, are commonly used. Many of these methods are also based on interpolation schemes for achieving sub-pixel accuracy. However, all of these algorithms implicitly assume that the laser spot shape is close to a circle. Further refinements of center-location algorithms consider embedding of the spot in noisy environments, fast-acquisition techniques, and multi-spot scenarios. However, to the best of our knowledge, there is no algorithm for processing spots which considerably deviate from circular/ellipsoidal shapes.

In this paper we use a conventional image moment method (IMM) for benchmarking the proposed RD-based algorithm. Although the above mentioned methods [16,20,21] may provide better accuracy for a centroid location, the advantages of IMM are its simplicity and performance. This method can be easy implemented for almost all platforms, including field-programmable gate arrays (FPGA). Moreover, in order to produce a high-performance algorithm, it is feasible to use an iterative process to locate the centroid of the laser spot. Using heuristic optimization techniques (e.g., Hill Climbing [22]), one can recalculate the centroid location after each step of the optimized algorithm. In this case there is no need for such a high accuracy.

The implementation of the image moment method is described below.

- An image acquired from a CCD camera is transformed into a single channel 8-bit gray image
- A simple thresholding is applied to produce a binary image. Each pixel with a value greater than $I_{th}$ is set to $2^{7}-1$
- Using the binary data we can find the outer contour of the spot. The topology information, such as nested contours, is skipped for the sake of simplicity
- Now we can calculate the center of mass of the area surrounded by this contour using the definition of the centroid:
  (3) $$\bar{x}=\frac{\sum_{i;j}x_{i}I_{ij}}{\sum_{i,j}I_{i;j}}\;\bar{y}=\frac{\sum_{i;j}y_{j}I_{ij}}{\sum_{i,j}I_{i;j}}$$
  where $I_{i,j}$ is an image matrix

**Figure 1:**
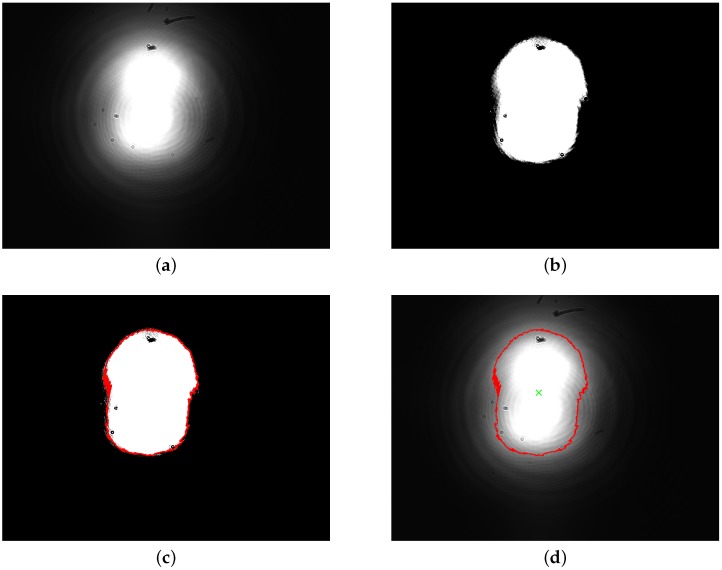
Steps of the image moment method applied to the original image *sample19*: (**a**) 8-bit single channel gray scale image; (**b**) binary image, obtained using the threshold value $I_{th}=2^{7}$; (**c**) contour detection using the binary data; (**d**) centroid calculation.

Figure 1 illustrates different stages of the above algorithm.

### 3.2. RD-FHN Laser Spot Centering

#### 3.2.1. Exemplification of the FHN-Based Computation

**Figure 2:**
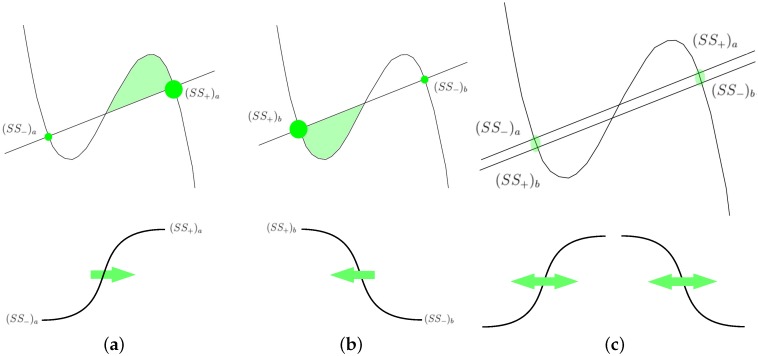
Nullcline representations for a bistable system. (**a**) bistable asymmetric configuration in which the second stable point is the most stable—therefore, in a system endowed with such a configuration, the wavefronts will always travel towards ${\left(SS_{+}\right)}_{a}$; (**b**) on the contrary, in this case, the first stable point is the most stable; (**c**) both asymmetric configurations are now superimposed, thus exposing the proximity of their stable points.

**Figure 3:**
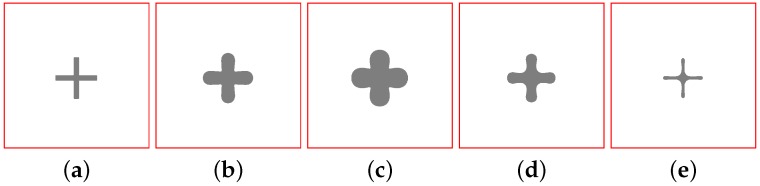
Model spatio-temporal evolution in a two-dimensional grid. (**a**) original pattern introduced as the initial condition by means of *u*; (**b**,**c**) the model is left to freely evolve for a limited number of iterations; (**d**,**e**) a switch of the nullclines configuration reverses the wavefront’s direction of propagation, and the original pattern contracts over itself.

The technique for calculating the coordinates of the laser spot by means of the FHN model uses the switch-of-phase mechanism introduced in [14,15] that analyses the nullclines in a bistable asymmetric configuration (see Figure 2). For a bistable nullcline configuration, it is possible to locally modify the relative stability of both stable points in order make one of them more stable than the other. In a broad description, the relative stability can be related to the area under the curve as shown in Figure 2a,b. The notation ${\left(SS_{\left\{+,-\right\}}\right)}_{\left\{a,b\right\}}$ denotes the most and least stable points respectively, for both configurations, *a* and *b*. Based on this, the following description aims to showcase the mechanism by which the RD model is used for computation:

- First, a two-dimensional integration grid, where each cell has a bistable nullcline configuration similar to the one depicted in Figure 2a is prepared. As initial conditions, the concentration values of u,v for all the grid points are set in the less stable state $SS_{-}$. As a result of this configuration, the system remains static in those levels of concentration for all cells in the integration grid.
- Then, a geometric figure is introduced in the model using u,v. This means that for a few cell points corresponding to the shape of the desired pattern, the values of u,v are set in $SS_{+}$. Consequently, a frontwave will be triggered, as can be seen in the series of Figure 3a–c, where the model is evolving from $SS_{-}$ towards $SS_{+}$. The diffusive term in Equation (2) explains this movement. Although the asymptotic behaviour is defined by the nullcline configuration, when a cell point changes its concentration levels it transfers this change to its neighbor’s by means of the diffusive link between them. Hence, the global result is a wavefront that moves the whole system towards $SS_{+}$.
- Depending of the particular algorithm used, the *END* condition for the current *phase* will be different. For instance, it can be stopped after a specific number of iterations, or wait until the model stops its evolution after reaching a static situation. Thus, in the previous step the spatio-temporal evolution was arbitrarily stopped after 1000 iterations.
- Finally, switching the nullcline configuration to the one depicted in Figure 2b produces a shift in the relative stability of the stable states. What in the previous step was $SS_{+}$ now becomes $SS_{-}$, and *vice versa*. This is possible due to the fact that the stable points in both configurations ${\left(SS\right)}_{\left\{a,b\right\}}$ are very close to each other, as depicted in Figure 2c, where both configurations were superimposed. Therefore, after a short period of adaptation, ${\left(SS_{-}\right)}_{a}$ becomes ${\left(SS_{+}\right)}_{b}$, and ${\left(SS_{+}\right)}_{a}$ becomes ${\left(SS_{-}\right)}_{b}$. As can be expected, the system starts evolving all its grid points towards the concentration values of u,v, corresponding to the new $SS_{+}$. In short, the original wavefront changes its direction of propagation. This process can be seen in the series of Figure 3c–e, where the model evolution was again stopped after 1000 iterations.

Needless to say, reversing the direction of propagation of the wavefronts will not have any other result than modifying the size of the original pattern that was introduced as initial conditions, as it has been shown in the whole series of pictures depicted in Figure 3. Therefore, in the current approach, endowing the system with computing capabilities means to encapsulate geometric operations in the wavefront propagation. In other words, it is necessary to find configurations for the FHN-model whose wavefront interactions can be related with specific geometric operations, instead of simply producing a change in the direction of propagation of the wavefronts. This fact turns out to be the cornerstone of the RD-based computation, at least in the approach that is exemplified above.

Herein, four different phases in correlation with four different model configurations are used. Based on the above description of the algorithm, each of these phases can be considered as a filter that performs a geometric operation over the original image. The succession of phases transforms a raw image coming from the camera into a small spot corresponding to the expected location of the laser spot center. Additionally, the spot size is increased in the last phase of the algorithm, to reach a desired size for the section of the laser beam.

#### 3.2.2. Thresholding Camera Data

The raw data coming from sensors has to be *adapted* somehow before being used in any algorithm. In the present case, the cameras provide a gray scale pictures with 8 bits. Besides, for the location of the laser center spot, only the information that represents laser light is relevant, because only such information (with respect to the current approach) will trigger the wavefronts used in the RD computation. Therefore, all information that does not represent laser light has to be filtered based on the particular value of a grayscale threshold.

**Figure 4:**
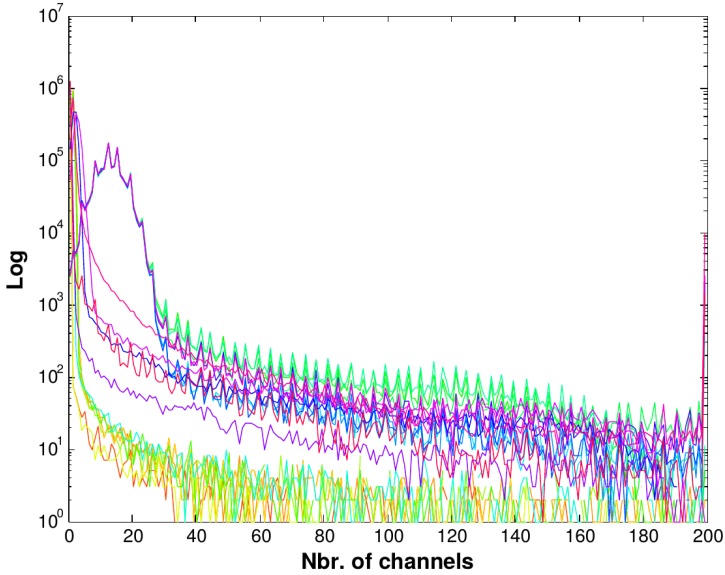
Overlaid histograms corresponding to the 22 samples. Notice that the Y-axis representing the number of pixels is in logarithmic scale.

The histograms for the 19 images that will be used in Section 4 are shown in Figure 4.

It can be seen that the majority of the information corresponding to the background is below 30 in the abscissa. This is approximately the starting point from which the plateau of the histogram starts for all the images. Values above this threshold represent laser light. The histogram also acts as a calibration method for both cameras and the RD algorithm.

#### 3.2.3. Algorithm Description

**Figure 5:**
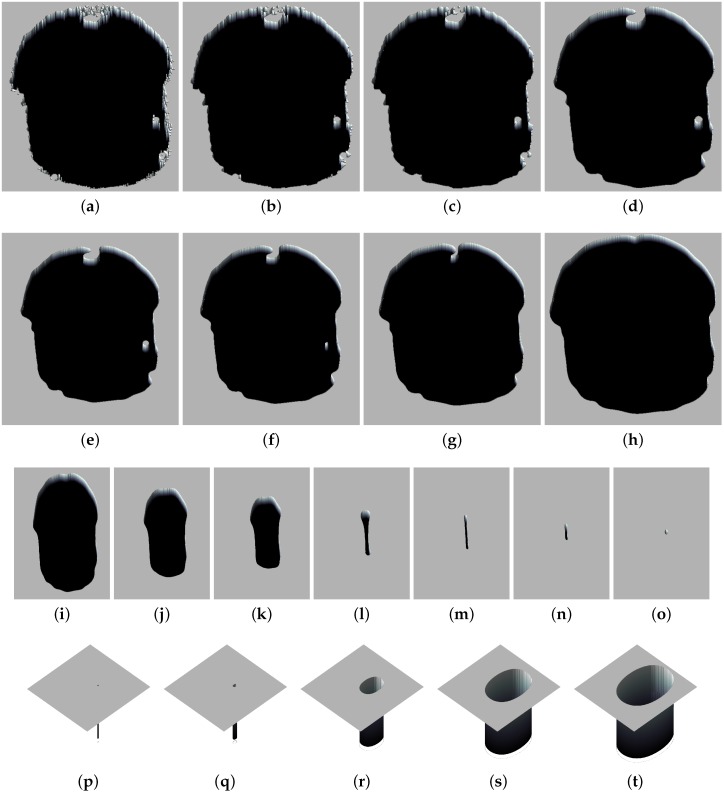
Steps of the RD-based laser spot algorithm applied to the image *sample19*: (**a**–**d**) first stage of the algorithm, where a contraction phase smoothes all of the noisy contour; (**e**–**h**) second stage, where an expansion phase removes undesirable isolated spots that are interpreted as noise; (**i**–**o**) third stage where an cotraction phase reduces its size until the supposed laser spot center; (**p**–**t**) final and extra stage that reconstructs the expected circular beam.

As described in Section 2, the FHN model accepts external information by simply using the initial concentration values of the state variables u,v. This will introduce a “pattern” in the system, as discussed in Section 3.2.1. Then, the system will evolve and clearly will overwrite such a pattern by the new u,v values. The evolution will be different in regards to the particular model configuration. In the present case, the “pattern” to be introduced corresponds the profile of the laser beam that will be transferred at the first stage of the algorithm using the *u* variable. For that purpose, the matrix containing the camera data has to be mapped from the original $\left[30\;200\right]$. After that, by means of the previously described *switch-of-phase* mechanism, the algorithm will perform smooth transitions from one *phase* to the next. Also, when a specific *phase* of the algorithm relies on a nullcline configuration similar to the one depicted in Figure 2a, it will be called *expansion-phase*, whilst the nullclines depicted in Figure 2b will correspond to a *contraction-phase*. This terminology was first introduced in [23], and it has been maintained in subsequent publications for the sake of clarity. The different stages of the FHN-based laser spot centering algorithm are described below:

- Firstly, a contraction phase where the result of the model evolution will be removal of the background noise. Besides, the edges of the beam section become smoother. An exemplification of this process is depicted in the series of pictures in Figure 5a–d. This phase runs for only 1500 iterations, a value that was determined experimentally.
- The second phase, an expansion in this case, removes all the remaining patches of wavefronts that appear as isolated regions and also further smooths the contour of the laser beam. This process can be seen in the series of pictures depicted in Figure 5e–h. In this case, 5000 iterations is the appropriate value.
- The third phase is again a contraction, where the wavefront representing the laser beam contour retracts over itself, decreasing its size until the supposed laser spot center. The result of this phase is a small spot that coincides with the centering of the laser beam. The process is shown in the series of pictures depicted in Figure 5i–o. The end condition of this phase is determined by the system reaching a static situation in the model evolution. The main reason underlying this behaviour is the constant speed of the wavefront propagation; therefore, the velocity of contraction is the same in all directions.
- A final expansion phase results in the growth of the small spot that was achieved in the previous step until it reaches the size of the supposed original laser beam contour. Although this phase is non-essential for obtaining the coordinates of the laser spot, it shows how the FHN-based computational approach is able to process geometric information. The process is represented in the last series of pictures shown in Figure 5p–t. In this last case, the number of iterations varies according to the desired size of the beam section, from which it is also trivial to extract the beam contour.

It must be noticed that all images in Figure 5 have been cut out around the area of interest to include a larger number of them in the figure. Also the chosen representation aims to give a 3D glimpse of what is actually a 2D grid, which makes it easier to see the RD evolution, but also slightly deforms the final perspective.

The set of FHN model values for each phase is represented in Table 1.

## 4. Results and Discussion

**Figure 6:**
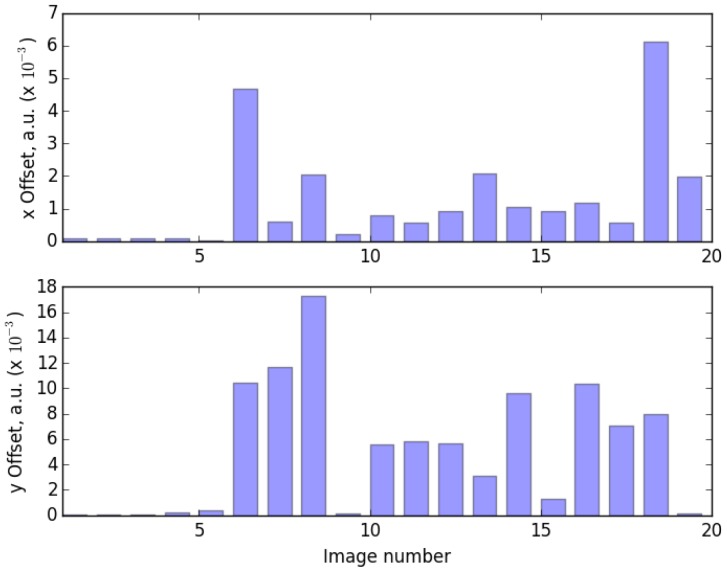
Illustration of the results depicted in Table 2. The height of each bar corresponds to the distance between centroids calculated by both methods.

In our experience, selection of the right metrics is a cumbersome task when trying to compare the outcome of a RD-based algorithm with the results of a standard one. However, in this work, the offset vector between centroids calculated by IMM and FHN-based method has been chosen. Since the same images were used as an input for both calculations, such vector being normalized to the number of pixels in each direction provides a meaningful metrics. The results are summarized in Table 2 which shows a good agreement between the standard image moment method and its FHN-based counterpart. Figure 6 depicts the results in terms of distances between centroids calculated by both methods.

It is remarkable that many of the traditional center-location algorithms tacitly assume that the laser spot outer contour can be approximated to a circle or an ellipse. In some cases, the beam is intentionally defocused in order to fit a Gaussian profile (whose contour maps are also circles—See for instance [16]), which may introduce large errors in the center position of non-symmetrical spots. On another hand, while using ellipses to approximate the shape of the spot contour may improve the calculation, one would expect the algorithm to produce non-neglectable errors for non-symmetrical spots as well.

Regarding the RD-based method, it is noteworthy that no fine tuning at all was necessary in any phase of the algorithm, in any case. As long as the threshold value obtained from a collection of samples is well defined, the algorithm works seamlessly for finding the center in all cases. Indeed, three samples were removed from the collection used in the comparison, as the IMM method was not able to properly handle those samples. However, this fact is not really relevant, because the IMM algorithm can be improved as much as necessary in regards to the particular optical set up (beam path, optical elements, *etc.*) that produces the misalignments, or can also be combined with other algorithms to increase its accuracy. It is, however, worth mentioning the robustness of the FHN-based solution. This quality makes the algorithm especially interesting for being integrated in other systems, for instance in an automatic alignment setup, where the robustness of the method (understood as the fact that a solution will be always provided) is preferable over accuracy.

## 5. Conclusions and Future Work

The proposed algorithm can accurately find the center of a spot, regardless of its shape or whether the spot image is highly noisy. As shown in the previous section, the algorithm behaves very well when the images are noisy, and when the spot presents strong arbitrary aberrations. In this context, the RD-based method may be useful during the calibration of beams in research facilities, or in data post-processing, wherein robustness of the method is preferred.

From a broader perspective, the RD-based computation has been in the spotlight of many works regarding non-linear science for a long time, due to the numerous and interesting properties exhibited by the spatio-temporal evolution of the models. The main problem faced by RD computation was the lack of stable states as the outcome of the computation. This stems from the fact that using excitable nullcline configurations has been the norm so far for implementing RD-based computation. However, such configurations only generate transient states that have to be checked iteratively, a fact that undermines the usefulness of the algorithm. The herein-presented approach based on the switch-of-phase mechanism in a bistable asymmetric nullcline configuration overcomes that problem, generating final stable states as the output of the computation.

Furthermore, the presented results are an extension of our previous studies, where different behaviors have been successively included in a RD-based computational core. The addition of the laser spot detection algorithm adds new capabilities to that novel approach, and represents another step in the direction of consolidating a computational framework completely based in RD computation. For that reason, it will be interesting to continue pursuing applications for the developed framework, to extend the range of applicability, and also with the aim of demonstrating the feasibility of using RD models to develop general purpose computation. This will also make interesting a port to specialized hardware.

## Figures and Tables

**Table 1:** Set of values for the FitzHugh–Nagumo (FHN) model, in order to reproduce the specific behaviors in each of the four stages of the Reaction Diffusion (RD)-based algorithm.

		FHN Parameters				
		α	β	ϵ	$D_{u}$	$D_{v}$
**Phase**	*contraction*	5	0.1	10	0.1	1.5
	*expansion*	4	−0.5	40	0.45	2
	*contraction*	5	0.1	10	0.1	1.5
	*expansion*	5	−0.2	10	0.1	1.5

**Table 2:** The comparison between the image moment method (IMM) and the RD method. Coordinates are in pixels. The offset value is normalized to the number of pixels in each direction.

		Centroid Coordinates					
		IMM x	IMM y	RD x	RD y	Offset x ($10^{-3}$)	Offset y ($10^{-3}$)
**Image**	*1*	342.330	584.317	342.452	584.337	−0.095	−0.021
	*2*	353.200	590.375	353.304	590.349	−0.081	0.027
	*3*	342.330	584.317	342.452	584.337	−0.095	−0.021
	*4*	366.411	585.350	366.306	585.524	0.082	−0.181
	*5*	373.349	594.326	373.339	594.661	0.008	−0.349
	*6*	568.699	552.488	562.710	562.493	4.679	−10.422
	*7*	649.642	536.352	648.890	547.549	0.588	−11.664
	*8*	646.670	599.226	644.034	615.806	2.059	−17.271
	*9*	131.899	460.435	132.180	460.296	−0.220	0.145
	*10*	662.283	453.218	661.256	458.574	0.802	−5.579
	*11*	662.263	453.169	661.517	458.714	0.583	−5.776
	*12*	662.642	453.075	661.465	458.493	0.920	−5.644
	*13*	213.383	477.399	216.049	480.340	−2.083	−3.064
	*14*	655.300	510.910	653.975	520.126	1.035	−9.600
	*15*	187.473	472.137	188.648	473.333	−0.918	−1.246
	*16*	655.563	511.081	654.045	520.999	1.186	−10.331
	*17*	625.730	463.174	624.991	469.963	0.577	−7.072
	*18*	578.307	531.479	586.126	523.829	−6.109	7.969
	*19*	574.611	380.773	577.164	380.872	-1.995	-0.103
